# Early-Life Risk Factors and Clinical Features of Food Allergy Among Thai Children

**DOI:** 10.1155/2024/6767537

**Published:** 2024-10-14

**Authors:** Samkhwan Thongsukkaeo, Yiwa Suksawat

**Affiliations:** ^1^Department of Pediatrics, Phramongkutklao Hospital and Phramongkutklao College of Medicine, Bangkok 10400, Thailand; ^2^Division of Allergy and Immunology, Department of Pediatrics, Phramongkutklao Hospital and Phramongkutklao College of Medicine, 315 Rajavithi Road, Ratchathewi, Bangkok 10400, Thailand

## Abstract

**Background:** Food allergy affects 1%–10% of children under five worldwide, with genetic and early-life factors playing a primary role. Reported factors include a family history of allergic diseases, personal atopic dermatitis, cesarean section, dietary restrictions during pregnancy and lactation, and the timing of introducing solid foods. This study was aimed at identifying various factors associated with food allergy and evaluate each food allergy's clinical features.

**Methods:** We conducted a case–control study with a participant ratio of 1:2 between cases and controls. Data were gathered from both groups of participants, and questionnaires included living area, sex, and natal history (birth details, maternal diet during pregnancy and breastfeeding, feeding history during infancy, family history of atopic diseases, and household smoking).

**Results:** All 72 cases with food allergy and 145 controls were included in the study. Term birth comprised a protective factor for developing food allergy (adjusted odds ratio [aOR] 0.213, *p* value 0.022). In contrast, personal atopic dermatitis (aOR 20.097, *p* value 0.001) and a family history of allergic disease constituted risks (aOR 3.183, *p* value 0.002). Food allergy was unrelated to cesarean section, low birth weight, dietary restrictions during lactation and pregnancy, exclusive breastfeeding, or the early introduction of complementary foods. The three most common food allergens were egg white (40.2%), wheat (34.7%), and cow's milk (30.5%), respectively.

**Conclusions:** In this study, risk factors associated with food allergy comprised a personal history of atopic dermatitis and a family allergic disease, which may be used as predictive factors for developing food allergy among Thai children.

## 1. Introduction

Food allergy affects approximately 1–10% of children under the age of five worldwide [1]. Currently, most available data is based on patients' self-reporting, which overestimates the prevalence of food allergy. This could result in patients or parents misinterpreting other adverse reactions to food, particularly food intolerance. Food allergy can be classified based on the immunologic response as IgE-mediated, non-IgE-mediated, or mixed IgE and non-IgE-mediated. The assessment of patients with food allergy uses a variety of surrogates, including clinical history and reliable diagnostic tests. Skin testing or serum food-specific IgE can be used to evaluate IgE-mediated food allergies. However, most patients with non-IgE-mediated food allergy should be confirmed by oral food challenge (OFC) test. The accuracy of food allergy diagnosis is essential in determining management and planning treatment.

The prevalence and pattern of food allergy considerably differ in each Asian country. In Thailand, the overall prevalence of food allergy in children aged 3–7 years is 1.1%, as confirmed by OFC [2], while in Japan, it reaches as high as 7.4% among boys and 6.3% among girls [3]. Food allergy often develops in the first few years of life, and cow's milk and egg are the most common causes among young children in most Asian countries [4]. In Thailand, shellfish allergy is more frequent among older children [5].

Genetic and early-life environmental factors are prominent risks for food allergy. Previously reported factors associated with developing a food allergy include a family history of allergic diseases, personal history of atopic dermatitis (AD), cesarean section, and environmental factors such as dietary restriction during pregnancy and lactation and the timing of introducing solid foods. However, preterm birth or exclusive breastfeeding might be protective factors for developing food allergy [6].

This study was aimed at identifying the various factors associated with food allergy among Thai children under the age of 18 and evaluating the clinical features of each food allergy. Because genetic potential and child-rearing in Asian populations can vary from those in Western countries, the risk and protective factors may differ from those identified in earlier studies [7].

## 2. Materials and Methods

We conducted a case–control study among children below 18 years from the Pediatric Department of Phramongkutklao Hospital, Thailand, between October 2021 and November 2022, including children with definite food allergy and age-matched controls with no history of food allergy. We aimed to have a ratio of at least 1:2 between cases and controls. When the history suggested IgE-mediated food allergy, the participants were assessed with the skin prick test (SPT), specific IgE, and food challenge when indicated. The SPT used commercially available extracts of common food allergens (ALK-Abelló, New York, United States), including egg white, egg yolk, cow's milk, soy, wheat, shrimp, crab, oyster, and clam. When allergen extracts were unavailable, the prick-to-prick (PTP) skin test using selected cooked foods was performed. SPT was performed by a trained medical professional using a lancet on the back or forearm. Histamine (10 mg/mL) and 50% glycerin were used as the positive and negative controls. An immediate reaction (wheal and erythema) was read after 15 min. Positivity for SPT was defined with a wheal diameter of at least 3 mm larger than that of the negative control. The specific IgE to each specific causative food was measured using the ImmunoCAP system (Thermo Fisher, Uppsala, Sweden), and a specific IgE level greater than 0.35 kUa/L was considered positive.

When SPT result or specific IgE was inconsistent with the patient's history or participants with a history of non-IgE-mediated food allergy, the next step would be an OFC. Open OFC tests were conducted in the hospital under the supervision of an allergist and trained staff with close observation for any adverse signs and symptoms. The OFC protocol was adapted from a standard recommended protocol [8]. Tests were performed when the children were completely well and had discontinued any antihistamine medication for at least 7 days before the challenge. Positive challenges were defined when patients developed significant symptoms and signs in one or more organs: cutaneous, respiratory, cardiovascular, and gastrointestinal systems.

The following criteria, as defined by EAACI [9], were used to identify definite food allergy: (1) a history of skin, respiratory, or gastrointestinal symptoms caused by consumption of a specific food and/or (2) positive specific IgE and/or SPT and/or (3) positive OFC.

Data were gathered from both groups of participants. The questionnaires included living area, sex, pre-/peril, and postnatal history (birth detail, maternal diet during pregnancy, maternal diet during breastfeeding, feeding history during infancy, personal history of allergic diseases, family history of any atopic diseases, and presence of household smoking). The diagnosis of allergic rhinitis was defined according to the ARIA guidelines as having two or more of the following symptoms: rhinorrhea, nasal congestion, sneezing, or nasal itching [10]. For the food allergy group, we collected data on the causative foods, the age of onset, detailed symptoms, and the test results. Written informed consent was obtained from the parents of all participants.

### 2.1. Statistical Analysis

All statistical analyses were performed using Stata 17 (StataCorp, College Station, Texas, United States). We described categorical variables with frequency and percentage and continuous variables with mean and standard deviation, or median and interquartile range, as appropriate. Fisher's exact probability test was used to identify the significant differences in categorical variables between cases and controls. An independent *t*-test or Mann–Whitney *U* test was used to compare continuous variables between cases and controls. Multivariate analysis was performed using logistic regression analysis, and a *p* value less than 0.05 was considered statistically significant.

## 3. Results

All 72 cases with confirmed food allergy and 145 controls completed the questionnaires. The overall ratio of cases to controls was 1:2.  Table 1 presents the demographic data for children with food allergy and the control group. There were no significant differences in sex, birth weight, types of milk ingested after birth, or household smokers between the two groups. The majority of participants were delivered by cesarean section, and the numbers between both groups did not differ significantly.

Data revealed significant differences between the two groups regarding gestational age and the age at which complementary foods were introduced (*p* < 0.05). The group with food allergy exhibited a higher prevalence of personal allergic diseases (52.8% vs. 11%; *p* < 0.001) compared to the control group. Among children with food allergy, 43% had a history of AD, whereas only 4.8% of children are without food allergy. Notably, there was a distinction in maternal nutrition between cases and controls. Maternal dietary restriction during pregnancy was associated with food allergy (*p* < 0.05), while dietary restriction during lactation exhibited no significant difference between the cases and controls. Additionally, a higher proportion of children with food allergy had a family history of atopic diseases. Moreover, there was a strong association between maternal allergic rhinoconjunctivitis (ARC) and children with food allergy (*p* < 0.001).

Table 2 shows early-life risk factors for developing food allergy. The multivariable analysis included cesarean section, term birth, birth weight, exclusive breastfeeding, age when supplementary food was introduced, personal or family of atopic disease, and maternal diet restriction during pregnancy or lactation. We found a significantly strong association between personal AD and an increased risk of food allergy in this study (adjusted odds ratio [aOR] = 20.097, 95% CI 7.168–56.348). Another factor associated with food allergy in this analysis was a family history of allergic diseases (aOR = 4.284, 95% CI 1.9–9.659). However, term birth was found to reduce the risk for food allergy (aOR = 0.213, 95% CI 0.57–0.8).

**Figure 1:**
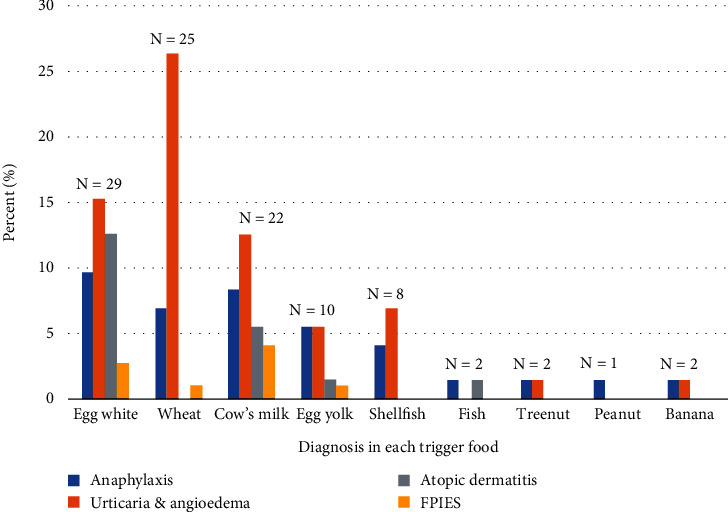
Number of children with food allergy categorized by type of food and disorder (*N* = 72). FPIES, food protein–induced enterocolitis syndrome.

Figure 1 displays the distribution of children with food allergy, categorized by the type of food allergen and the food allergy disorder. The study identified three prevalent food allergens: egg white (40.2%), wheat (34.7%), and cow's milk (30.5%). Nut allergy typically manifests between 21 and 24 months of age. Most patients reacted to a single food (65.2%), while 34.8% reacted to multiple foods. Notably, this study reveals that urticaria was the most common food disorder, followed by anaphylaxis and AD. Specifically, individuals with egg white allergy frequently showed symptoms of AD. Wheat was the primary allergen associated with urticaria. Anaphylaxis occurred more frequently in patients with egg white allergy, while cardiovascular symptoms were not observed in any children. Gastrointestinal symptoms were more predominant among cow's milk allergic patients than among patients with other food allergies. Approximately 25%–30% of patients with IgE-mediated egg white, wheat, or cow's milk allergy required an OFC for a definitive diagnosis.

## 4. Discussion

This study showed that personal AD and a family history of atopy were associated with an increased risk of food allergy, whereas term birth was associated with a reduced risk of food allergy.

Preterm birth should be considered a risk factor for food allergy because food antigen absorption is significantly increased in the gastrointestinal tract during the immaturity of the mucosal barrier. However, the prevalence of preterm infants in our study was lower than that in a relevant study [6], suggesting potential distinctions in our results. Low birth weight, often linked to insufficient secretory IgA and digestive system immaturity, theoretically facilitates increased absorption of food proteins, potentially leading to the later development of food allergies. Surprisingly, our findings did not reveal any significant association between low birth weight and the likelihood of developing food allergies. Consequently, the hypothesis positing an immature gut mucosa causing increased permeability to large food proteins, predisposing the infants to early sensitization, should be questioned. Our study highlights that term infants may have a reduced risk of developing food allergies. According to related research, the gastrointestinal tract's ability to eliminate allergenic food proteins improves with gestational age [11]. Additionally, the gastrointestinal tract in term infants is more mature and has more developed gut microbiota compared to that in preterm infants [12]. However, due to the small sample size of the study, it is challenging to definitively conclude that term birth is a protective factor, despite the observed aOR of 0.213.

Children with food allergies have gut microbial compositions different from those without. Early-life exposure to various microorganisms, such as mode of delivery, might impact the developing immune system. Babies delivered via cesarean section lack exposure to their mother's vaginal and rectal microorganisms, a crucial process that has been implicated in increasing the risk of allergic diseases in studies. Currently, cesarean section rates are rising worldwide, and the cesarean section rate in our hospital from 2021 to 2022 was approximately 30%. Due to the high number of cesarean sections in both groups, our study revealed insufficient evidence that the mode of birth affects a child's risk of developing food allergy.

Exclusive breastfeeding during infancy did not affect the development of food allergy, which was the same result of a recent systematic review and meta-analysis [13]. Despite any potential influence on allergy risk, breastfeeding remains the most recommended feeding practice for infants due to its numerous health benefits. Unfortunately, the findings of population-based research conducted in recent years concerning allergy risk remain controversial. Genetic, environmental, and nutritional factors may influence the impact.

With conflicting information and suggestions from many authoritative organizations, the optimal age for introducing solid feeding remains to be determined. The World Health Organization suggested 6 months of exclusive breastfeeding and the introduction of supplementary food [14]. Some randomized controlled studies of introducing allergenic solid foods revealed a reduced incidence of food sensitization and food allergy when taken early, often between the ages of 4 and 6 months [15–17]. Our investigation revealed that the timing of initiating complementary food did not significantly differ between the two groups, with most participants commencing after 6 months. Moreover, our study did not show any discernible benefits in reducing the risk of food allergies among children introduced to complementary food before 6 months. In Thailand, a prevalent practice among parents is to initiate complementary foods for their children around the age of 6 months, driven by concerns about aspiration issues. The *Mother and Child Health Care Handbook* in Thailand also recommends introducing supplementary food at 6 months. It is important to note that, apart from early peanut introduction for infants aged 4–11 months with severe eczema or egg allergy, insufficient clear evidence supports the notion that the timing of food introduction can lower the prevalence of food allergy [15]. Peanut consumption is closely related to the prevalence of peanut allergy, and it varies significantly between countries. In countries with low peanut consumption and low peanut allergy, such as Thailand, the recommendation for peanut introduction is based on current dietary practices.

Interestingly, the most relevant findings in this study were that food allergy was strongly associated with personal allergic diseases, especially AD, and allergic diseases in the family. These findings revealed that developing food allergy was strongly related to genetic factors and atopic features. Lack's dual-allergen exposure theory postulated that low-dose cutaneous exposure to food allergens was a risk factor for food allergy. In contrast, early ingestion of allergens may result in oral tolerance [18]. When the skin barrier is disrupted, such as in AD, skin exposure to food allergens might be more significant, which might explain why food allergy and AD frequently coexist.

Families with a history of allergies also had an increased risk of food allergy among their children [19]. Our result showed that maternal ARC was significantly associated with children with food allergy (*p* value < 0.001). This result supported the findings in related studies and provided additional evidence that a family allergy history and a child with allergic comorbidity might significantly impact the development food allergy. As a result, these two risk factors may be used as biomarkers to predict developing food allergy among infants.

Although several factors have been reported to be associated with risk factors for food allergy, they failed to show statistical significance in our study. Maternal diet restriction during pregnancy and lactation were more likely to develop food allergy among their children [20]. Due to the small number of pregnant and lactating mothers (*n* = 12) and the limited sample size of our study, the magnitude of the effect of maternal food restriction during pregnancy and lactation was large but not statistically significant. Several systematic reviews and randomized trials have suggested no benefits from restricting common allergenic foods among pregnant and lactation mothers [21]. Moreover, the American Academy of Pediatrics has concluded that insufficient evidence supports maternal dietary restriction during pregnancy and lactation prevention strategy for atopic disease [22].

In our study, egg white and wheat were the most common food allergens. According to the data from the GUSTO cohort, the most common food allergens among preschool-aged children were egg, followed by cow's milk. Wheat was uncommon in this population. However, similar to our results, shellfish was the predominant food allergen in the older age group [23]. Based on the national survey on immediate food allergy conducted in Japan, the most frequent causative foods were hen's egg, cow's milk, and wheat [24]. Therefore, further study is required to ascertain regional differences regarding the prevalence of food allergy in Asia. Although food protein–induced allergic proctocolitis (FPIAP) stands out as one of the most common food allergic disorders encountered in outpatient departments, it was intentionally omitted from this study. The exclusion was necessitated by the fact that patients did not undergo confirmation of their diagnosis through an OFC test. Parents, possibly apprehensive about bloody mucous diarrhea, opted to avoid the potentially causative food and declined participation in the test.

The strength of this study is the standard clinical evaluation of patients suspected of having food allergy, including reliable history, followed by in vivo/in vitro IgE for specific food in IgE or mixed-type food allergy and an OFC to confirm the diagnosis. We included patients reporting current food allergy and those diagnosed with any food allergy disorder.

Our study encountered potential limitations. First, the retrospective recall for various exposures, such as the age of each food introduction or the type of milk ingestion after birth, may have resulted in recall bias. However, recall error is much less likely for demographic factors such as personal allergy history or family history of allergic disease. Second, some information parents could not remember was obtained from an electronic medical database. However, several patients with missing data were excluded from the study, which may have introduced selection bias. Third, the study size was small, so some risk factors might not be adequately powered to identify statistical significance.

## 5. Conclusion

In conclusion, our study found that children's early-life risk factors for developing food allergy were a personal history of AD and a family history of atopic disease, while term birth was a preventive factor for food allergy. Due to limitations in the study design, further prospective clinical studies are needed to confirm other factors associated with developing food allergy.

## Figures and Tables

**Table 1:** Demographic data between children with and without food allergy (control).

	**Children with food allergy, ** **N** ** = 72**	**Control, ** **N** ** = 145**	**p** ** value**
Sex			0.598
Female	37 (51.4%)	69 (47.5%)	
Median age, age range (months)	10	20	
0–12	43 (59.7%)	57 (39.3%)	
13–24	18 (25%)	25 (17.2%)	
25–36	3 (4.1%)	17 (11.7%)	
37–60	1 (1.4%)	28 (19.4%)	
61–180	7 (9.8%)	18 (12.5%)	
Mode of delivery			0.093
Cesarean section	31 (43%)	80 (55.1%)	
Gestational age (weeks)			0.020^∗^
< 37 weeks	4 (5.55%)	28 (19.3%)	
37–40 weeks	62 (86.1%)	110 (75.8%)	
> 40 weeks	6 (8.35%)	7 (4.9%)	
Birth weight (g)			0.109
< 1500 g	0	7 (4.8%)	
1500–2499 g	6 (8.3%)	25 (17.2%)	
2500–4000 g	64 (88.8%)	111 (76.6%)	
> 4000 g	2 (2.9%)	2 (1.4%)	
Type of milk ingestion			0.283
Exclusive breastfeeding	36 (50%)	58 (40%)	
Partial breastfeeding	30 (41.7%)	77 (53.1%)	
Formula feeding	6 (8.3%)	10 (6.9%)	
Age at introduction of complementary food (months)			0.012^∗^
≤ 6 months	58 (80.6%)	116 (80%)	
Personal allergy history^a^			< 0.001^∗^
None	34 (47.2%)	129 (89%)	
Asthma	1 (1.4%)	6 (4.1%)	
ARC	5 (6.9%)	3 (2.1%)	
AD	31 (43%)	7 (4.8%)	
Household smoker	18 (25%)	49 (33.8%)	0.187
Dietary restrictions during pregnancy	6 (8.3%)	3 (2%)	0.029^∗^
Dietary restriction during lactation	6 (8.3%)	4 (2.8%)	0.065
Family history of allergic disease^a^			
Paternal asthma	2 (2.7%)	3 (2%)	0.743
Maternal asthma	4 (5.6%)	3 (2%)	0.171
Paternal ARC	13 (18%)	13 (8.9%)	0.052
Maternal ARC	21 (29.2%)	14 (9.7%)	< 0.001^∗^
Paternal AD	2 (2.7%)	0	NA
Maternal AD	2 (2.7%)	1 (0.7%)	0.215

*Note:* Values are presented as numbers (percent). Partial breastfeeding combines breastfeeding and formula feeding.

Abbreviations: AD, atopic dermatitis; ARC, allergic rhinoconjunctivitis.

^a^Sample was able to choose more than one underlying disease.

^∗^Statistically significant.

**Table 2:** Factors associated with food allergy from univariate and multivariate logistic regression.

**Parameters in models**	**Univariable** ^**a**^			**Multivariable** ^**a**^		
	**aOR**	**95% CI**	**p** ** value**	**aOR**	**95% CI**	**p** ** value**
Term infant	0.253	0.085–0.756	0.014^∗^	0.213	0.57–0.8	0.022^∗^
Low birth weight	0.325	0.129–0.820	0.017^∗^	0.871	0.224–3.382	0.842
Personal atopic dermatitis	16.803	6.811–41.453	< 0.001^∗^	20.097	7.168–56.348	< 0.001^∗^
Dietary restrictions during pregnancy	4.303	1.044–17.737	0.043^∗^	6.74	0.668–67.985	0.106
Family history of allergic disease	3.281	1.818–5.922	< 0.001^∗^	4.284	1.9–9.659	< 0.001^∗^

*Note:* Term infant, who was born at gestational age of 37–40 weeks. Low birth weight, < 2500 g. Family history of allergic disease included paternal, maternal, and sibling among first-degree relatives.

Abbreviations: aOR, adjusted odds ratio; CI, confidence interval.

^a^Comparison of children with food allergy group versus control group.

^∗^Statistical significance.

## Data Availability

The data used to support the findings of this study are included in the article.
